# Implementation of a 3D Coupled Hydrodynamic and Contaminant Fate Model for PCDD/Fs in Thau Lagoon (France): The Importance of Atmospheric Sources of Contamination

**DOI:** 10.3390/ijerph7041467

**Published:** 2010-03-30

**Authors:** Sibylle Dueri, Dimitar Marinov, Annie Fiandrino, Jacek Tronczyński, José-Manuel Zaldívar

**Affiliations:** 1 European Commission, Joint Research Centre, Via E. Fermi 2749, 21027 Ispra (VA), Italy; E-Mails: sibylle.dueri@ird.fr (S.D.); dimitar.marinov@jrc.ec.europa.eu (D.M.); 2 Ifremer, Laboratory of Environmental Resources, Bd Jean Monnet, BP 171, 34203 Sète, France; E-Mail: annie.fiandrino@ifremer.fr; 3 Ifremer, Laboratory of Biogeochemistry of Organic Contaminants, BP 21105, 44311 Nantes, France; E-Mail: Jacek.Tronczynski@ifremer.fr

**Keywords:** PCDD/F, Thau lagoon, POPs, fate model, hydrodynamic model

## Abstract

A 3D hydrodynamic and contaminant fate model was implemented for polychlorinated dibenzo-*p*-dioxins and dibenzofurans (PCDD/Fs) in Thau lagoon. The hydrodynamic model was tested against temperature and salinity measurements, while the contaminant fate model was assessed against available data collected at different stations inside the lagoon. The model results allow an assessment of the spatial and temporal variability of the distribution of contaminants in the lagoon, the seasonality of loads and the role of atmospheric deposition for the input of PCDD/Fs. The outcome suggests that air is an important source of PCDD/Fs for this ecosystem, therefore the monitoring of air pollution is very appropriate for assessing the inputs of these contaminants. These results call for the development of integrated environmental protection policies.

## 1. Introduction

Coastal lagoons are shallow water ecosystems characterized by high primary and secondary production, high biodiversity, and an elevated number of different habitats [1]. From a physical-hydrodynamic point of view, coastal lagoons are characterized by a high spatio-temporal variability and large fluctuation in physical and chemical conditions [2]. They are partly saline as a result of their proximity to coastal waters, but are also substantially influenced by freshwater flows. These complex, heterogeneous and highly productive ecosystems are areas of high economic importance due to their exploitation for aquaculture and touristic purposes. Furthermore, these habitats have a key role as nursery and rest areas for a great number of marine species and birds. However, during the last decades these ecosystems have been exposed to increasing anthropogenic pressures related to urban, domestic, agricultural and/or industrial effluents, as well as the exploitation for port activity, aquaculture and fishing. These pressures had increased the level of pollution and had contributed to the removal of indigenous species and changes in food web structure [3,4].

Polychlorinated dibenzo-*p*-dioxins (PCDDs) and dibenzofurans (PCDFs) are two groups of almost planar tricyclic aromatic compounds with a number of chlorine atoms that can vary between one and eight. These compounds do not occur naturally, but they are formed as by-products during the production of other chemicals or during combustion and incineration processes. The major sources of contamination by PCDDs and PCDFs include: industrial plants (iron, steel and non-ferrous metals plants), power plants (gas, oil, coal), waste incineration, road transport (fuel combustion) and mineral production. PCDDs and PCDFs are generally released into the air and transported through the atmosphere. These compounds have low solubility and are highly hydrophobic, thus they tend to accumulate in organisms and to adsorb to particles. Since they are very persistent they can be detected in almost every environmental compartment [5]. Of the 210 isomers, 17 that have chlorine atoms at least in the positions 2, 3, 7, 8 are especially toxic and persistent.

Along with field studies and monitoring activities, models are important tools to understand the fate and transport of contaminants and to assess their impacts on communities and ecosystems, especially in systems with high variability such as coastal lagoons [6]. In fact, the assessment of the response of an aquatic ecosystem to a contaminant requires the knowledge of the range of concentrations likely to be found and the temporal variability of the distribution. Since it is not possible to monitor at the necessary frequency and with the spatial sampling resolution required to capture the spatio-temporal variability typical of coastal lagoons, a modeling approach helps to complement experimental data and fill the gaps in the monitoring programs. Furthermore, modeling can support the testing of different management strategies to improve ecosystem state [7].

The objective of this study was to improve our understanding of the significance of different sources, pathways and processes affecting the partitioning, distribution and spatial-temporal variability of PCDD/Fs in the Thau lagoon (France). Therefore, we implemented a 3D coupled hydrodynamic-contaminant fate model and used experimental measurements of contaminant concentration in air, rivers and sediments as boundary conditions. The model was applied to calculate the variation of the concentrations and fluxes of PCDD/Fs in the lagoon and to estimate monthly and yearly exchange at the air-water and sediment-water interface. The main routes of PCDD/Fs contamination as well as their seasonal variability have been investigated.

## 2. Study Area: The Thau Lagoon (France)

**Figure 1.**
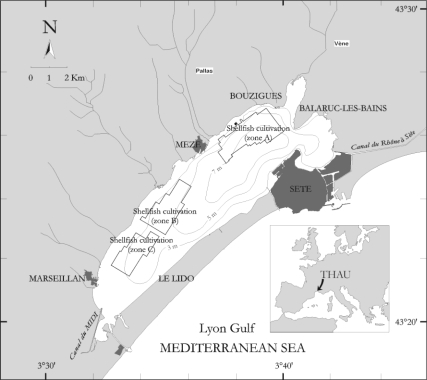
The Thau lagoon and its watershed. Connections with the Mediterranean Sea are located at the extremities: in the Sète city and south of Marseillan village.

Thau lagoon, located on the French Mediterranean coast, is a 25 km long, 5 km wide and on average 4 m deep coastal lagoon, which is sheltered and connected to the open sea with two narrow sea mouths (Figure 1). The catchment area is small (280 km^2^) and drained by numerous small streams with intermittent flows. The lagoon is characterized by a wide range of water temperatures and salinities varying from a temperature of 5 °C and a salinity of 27 (according to the Practical Salinity Scale 1978, PSS78) in February to a temperature of 29 °C and a salinity of 40 (PSS78) in August. Precipitation shows large interannual variation with minima of 200 and maxima of 1,000 mm per year. Wind is often strong with a mean of 118.5 days per year above Beaufort force 5 (data from Météo-France), particularly when it is blowing from the Northwest (the so called “Tramontane”).

Besides its ecological interest as a recruitment zone for some sea fish species, the lagoon is of notable economic importance due to shellfish cultivation, about 15,000 tons per year, which is amongst the highest in the Mediterranean Sea. During summer, the Thau lagoon may undergo anoxia that can lead to important economic losses [8].

During the last 20 years, the Thau lagoon has been extensively studied, with investigations of the exchange between the water column and sediments, the oysters farming activities, the impact of the watershed and interactions with the Mediterranean Sea [9–11]. Various numerical models have been developed, focusing on hydrodynamics [12], nitrogen and oxygen cycles [13,8], plankton ecosystem [14], impact of shellfish farming [15–17] and macrophytes [18–19]. However, none of the above mentioned models focus on the fate and effects of contaminants in the lagoon.

Some of the existing models have also been introduced into a decision support system to analyse the problem related with diffuse contamination by fecal bacteria [20]. This approach opens an integrated view of coastal zone management where the different options and management alternatives can be analyzed within the same framework [21]. In this context, the present contaminant fate model would be valuable as a tool for investigation/scenario making of persistent organic pollutants (POPs), in particular PCDD/Fs, for Thau lagoon.

## 3. Model Description and Implementation

A coupled 3D hydrodynamic, contaminant fate and ecological model was developed to simulate the pollutant concentrations in water bodies considering the exchange of contaminant with the atmosphere and the sediments. The model, which was originally implemented for the simulation of plant protection products in the Sacca di Goro lagoon, Italy [6], has been progressively extended for a variety of POPs studies involving the simulations of PCBs [22], PAHs [23] and presently PCDD/Fs. The contaminant fate module includes parameterization of all relevant physico-chemical properties and processes affecting the distribution and fluxes of POPs. Besides, the model is coupled with an ecological food-web module that considers the biomass variation of two phytoplankton populations, two zooplankton populations, bacteria and detritus [24]. As a result the tool is able to simulate the fate and distribution of contaminants considering physical, chemical and biological processes.

The model coupling was achieved by incorporating the two modules (contaminants fate and ecological) into the program structure of COHERENS model (COupled Hydrodynamical Ecological model for REgioNal Shelf seas) a 3D finite-difference multi-purpose model dedicated to coastal and shelf seas [25,26]. The hydrodynamic/physical part was kept unchanged while numerical solvers of the original model code were used for the new modules. In principle, COHERENS has been selected as a framework tool for the study of POPs that allows the coupling, development and integration of different sub-models. Moreover, COHERENS is freely available for scientific purposes, well documented [25] and it has been exhaustively verified, especially for test cases such as advection processes, turbulence closure schemes, plumes, river fronts, marine biological problems, *etc*. The complete model documentation together with program code can be requested through the web site (http://www.mumm.ac.be/EN/Models/Coherens/index.php).

### 3.1. Hydrodynamic Model

**Figure 2.**
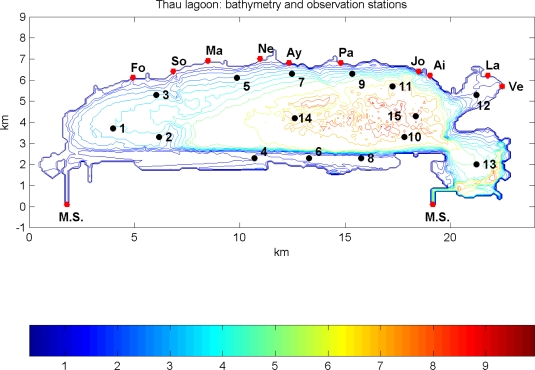
Thau lagoon bathymetry, inlets of streams and rivers, connections with Mediterranean Sea and selected observation stations.

The bathymetry and boundary limits of the Thau lagoon (France, N 43°25′03″ and E 3°36′70″) are presented in Figure 2 together with the locations of observation stations inside the lagoon, connections with Mediterranean Sea and river/stream inlets. The Thau lagoon 3D integrated model was build on a homogeneous spherical grid corresponding to horizontal 100 m by 100 m of cell size in Cartesian co-ordinate system. The vertical spatial resolution of the model respects the range of water depths in Thau lagoon—average depth circa 4.06 m (min = 0.7 m and max = 10 m)—and aiming to reduce the total CPU time the model was run with seven σ-vertical layers. The model time step, restricted by the spatial resolution and barotropic mode, was set up to 4 s. Under these conditions, a typical simulation run (one year, starting on 1st of January), including also the ecological module, takes around 10 days of CPU time on a Mac Pro1.1 with a dual core Intel Xeon processors of 2.66 GHz each.

The model was forced using meteorological data of air temperature at 2 m above the water surface, wind speed and direction at 10 m, precipitation rate, cloud cover, and relative humidity at Sète station. The data was provided by Meteo France (http://www.meteo.fr) for the two-year time period of 2004–2005. The model boundary conditions were defined by setting the watershed inputs and the exchange through the connections with open sea. The river fresh water discharges were specified on daily basis using SWAT watershed model output [11]. The open sea boundary conditions for temperature and salinity were obtained from linear interpolation between measured data sets. In addition, the nutrients at the connection with Mediterranean Sea were specified according to Plus *et al.* [18–19]. Finally, the hydrodynamic-physical module was initialized homogeneously with null current velocities, constant vertical profiles for temperature (T = 6 °C) and salinity (S = 35, PSS78).

### 3.2. Contaminant Fate Model

**Figure 3.**
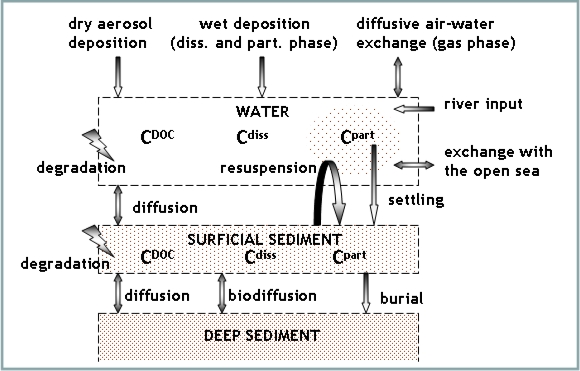
Schematic diagram of the processes represented in the contaminant fate model.

The contaminant fate model represents the main processes affecting the partitioning and distribution of PCDD/Fs in the water column and in the surface sediments, as presented in Figure 3. Thus, in the model, the total contaminant concentration comprises three different phases: freely dissolved concentration *C_diss_*, bound to dissolved organic carbon (DOC) *C_DOC_* and in the particulate phase *C_part_*. These concentrations are regulated by the exchange fluxes at the air-water and sediment-water interface as well as lateral inputs from rivers and exchange with the open sea. Atmospheric fluxes include dry and wet deposition as well as diffusive air-water exchange, while settling, resuspension and diffusive fluxes are the main processes occurring at the water-sediment interface. The sediment layer is subdivided in a surface sediment layer with changing contaminant concentrations and a deep sediment layer with fixed boundary concentrations. Between the two sediment sub-layers the model considers physical and biological diffusion as well as burial fluxes. Moreover the model considers degradation of contaminant in water and surface sediment. A detailed description of the equations and parameterization of the model is provided in the Supplementary Information Section.

To run the model, the boundary conditions representing the PCDD/Fs concentrations in the atmosphere, sediments, rivers and open sea have to be specified. During the experimental campaign carried out at Thau lagoon in May 2004 and November 2005 [27] concentrations in the air (particulate and gas), in river water, sediments, and in the water of the Thau lagoon were measured. Rainwater concentrations could not be collected during the sampling campaigns due to the absence of rainy events, therefore, in the model they had to be extrapolated from the correlation to gaseous and particulate air concentrations. Besides, for some congeners the concentrations in one or more boundary compartments were below the detection limit and therefore it was impossible to establish boundary conditions to run the model. Only PCDD/Fs having concentrations above the detection limits in air, sediment and river water were modeled and this applied to 12378-PeCDD, OCDD, 2378-TCDF, 12378-PeCDF, 23478-PeCDF, 123478-HxCDF, 123678-HxCDF, 234678-HxCDF and 123789-HxCDF. For simplicity the congeners having the same chlorination degree were grouped under one label: PeCDF assembles the concentrations of 12378-PeCDF and 23478-PeCDF while HxCDF represents the sum of 123478-HxCDF, 123678-HxCDF, 234678-HxCDF and 123789-HxCDF. Moreover, in the following text the position of chlorine atoms were omitted (see Table 1).

### 3.3. Ecological Model

To include the effect of organic matter dynamics on PCDD/Fs partitioning, the contaminant fate model was coupled with a previously developed ecological model [24], which we considered as the minimum model able to represent seasonal plankton dynamics. The model includes two phytoplankton compartments, two zooplankton compartments and a microbial loop representing bacteria and detritus. A detailed description of the model equations and parameters can be found in [24]. The coupling is performed through the calculation of the particulate organic carbon (POC) distribution in the water column, which is calculated as the sum of detritus, bacteria, phytoplankton and zooplankton expressed in mg C · m^−3^ [24]. In the present study, we did not consider toxic effects on plankton communities since there is no evidence of toxicity at the observed PCDD/Fs concentrations.

## 4. Model Performance Assessment

### 4.1. Physics and Hydrodynamics

A previous intercomparison study on hydrodynamic simulations for the Thau lagoon performed in DITTY project (http://www.dittyproject.org/) concerning currents directions and speeds showed that COHERENS and MARS3D, a model developed at IFREMER [12,28] reproduced adequately spatial and temporal dynamics of this coastal system producing almost similar results. Therefore, in this study the physical part of the COHERENS 3D model for Thau lagoon was assessed only for temperature and salinity fields. The present application was based on default k-ɛ turbulence scheme of COHERENS model [25] with heat transfer and water optical parameters set as follows: attenuation coefficient for infrared radiation 5.0 (m^−1^), infrared fraction of solar irradiance 0.5, slope parameter relating to salinity 0.05 (PSU^−1^ m^−1^), attenuation coefficient for PAR 1.0 (m^−1^) and exponential fraction of hyper-exponential PAR decay 0.25.

**Figure 4.**
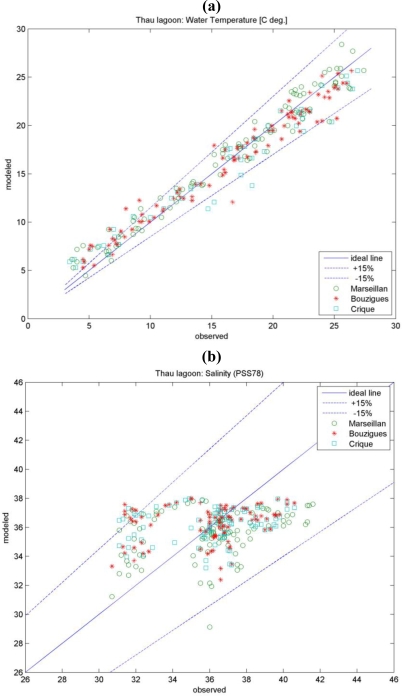
Comparison of surface layer temperature **(a)** and salinity **(b),** calculated by the model during 2004–2005 against field data (http://www.dittyproject.org/) for Marseilan (Station 3), Bouzigues (Station 15) and Crique (Station 12) zones in Thau lagoon.

**Figure 5.**
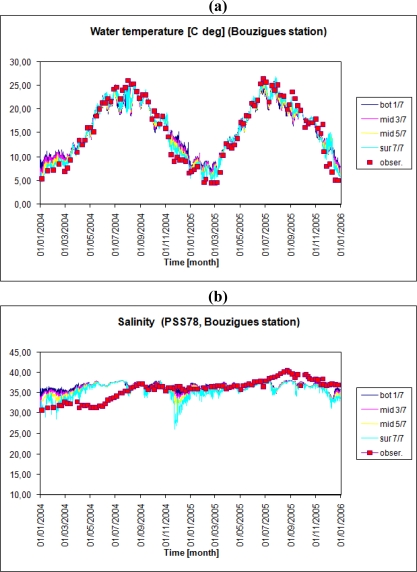
Annual temperature cycle **(a)** and salinity **(b)** calculated by the model with seven vertical layers (1 corresponds to the bottom layer, 7 to the surface) together with surface layer field measurements for deeper Bouzigues zone (Station 15) in Thau lagoon for 2004–2005.

The comparison of surface water layer temperatures calculated by the model during 2004–2005 against field data (http://www.dittyproject.org/) for Marseilan (Station 3), Bouzigues (Station 15) and Crique (Station 12) zones in Thau lagoon is presented in Figure 4a. In general, the model gives reasonably good results within ±15% error levels and the maximum deviations were found during temperature extremes, in particular the winter period, at the more shallow and/or sheltered parts of Thau lagoon located faraway from open sea connection (near to Station 3). In addition, the details about the annual temperature cycle calculated by the model are shown together with field measurements for deeper Bouzigues zone (Station 15) in Figure 5a. The vertical temperature stratification of water column could be observed annually but it is more evident during the cold seasons of the year.

Similarly, the comparison of surface water salinity calculated by the model against measurements in Thau lagoon for the above mentioned three stations also gives reasonable good results with a mean deviation range of ±15% (Figure 4b). The maximal deviations were observed either for periods of very low (or none) river input conditions or vice versa during the intensive fresh water discharge (Figure 5b). Both situations could be related to the watershed model output, serving as basis for setting up the boundary conditions of the 3D lagoon model. Thus, an improved performance of the watershed model, which was run with one day as time step, in terms of meteorological data and diel flow variability should certainly assure further amelioration of the lagoon model [29].

**Figure 6.**
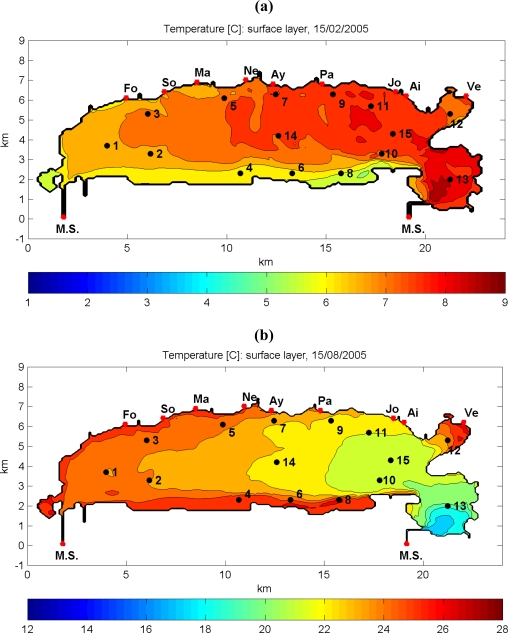
Spatial distribution maps of surface water temperatures in Thau lagoon for February **(a)** and August **(b)** 2005.

The spatial variability of surface water temperatures in Thau lagoon is presented in Figure 6 for winter (February) and summer (August) periods. The thermal regime of coastal lagoons is clearly represented in this figure, with higher temperature variances in comparison with open sea. In addition, due to its specific bathymetry and more restricted water circulation, the water masses in the periphery of Thau lagoon are characterized with extreme temperatures excursions almost for the entire year.

### 4.2. Contaminant Fate Model

**Figure 7.**
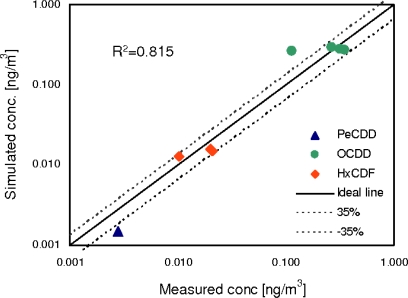
Comparison between simulated surface water bulk concentrations of PeCDD, OCDD and HxCDF and measurements [27] at Station 1, 13, 14 and 15 during sampling campaign (R^2^ = 0.815).

The amount of data available for PCDD/Fs in Thau lagoon water column [27] allows only a preliminary assessment of the contaminant fate model by comparing the simulated water concentration with the experimental concentrations measured during the sampling campaign (Figure 7). The model was run for PeCDD, OCDD, TCDF, PeCDF and HxCDF, for one year, beginning on the 1st of January 2005. Since TCDF and PeCDF concentrations in surface water were below the detection limit, the model performance could not be checked for those compounds. Simulated total concentration of PeCDD, OCDD and HxCDF were compared to concentrations measured at the sampling stations 1, 13, 14 and 15. From the comparison we can argue that the model results are in the range of observed concentrations because the average error is between ± 35 % of the measured value and the R^2^ is 0.815, p < 0.05. Even though only a preliminary assessment of the contaminant fate module was possible, the satisfactory performance of this module during previous studies with other contaminants [6,23], for which longer time series and a more complete datasets were available, supports its use for the investigation of the environmental fate of POPs.

## 5. Results and Discussion

**Figure 8.**
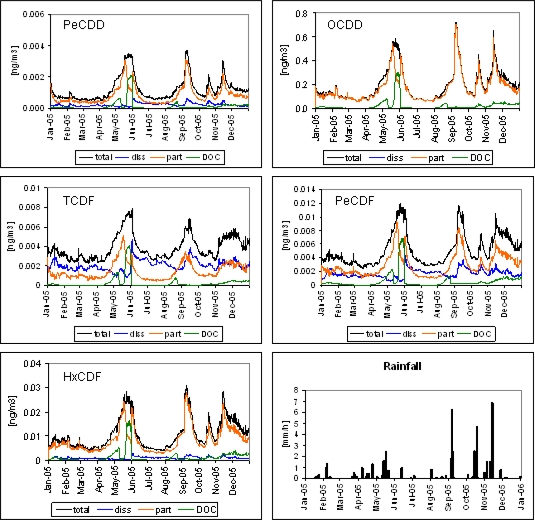
Simulated concentrations of PeCDD, OCDD, TCDF, PeCDF and HxCDF at Station 14 during 2005 and rainfall registered in Sète meteorological station.

Due to their hydrophobicity, most of the PCDD/Fs compounds show a high affinity to the particulate fraction. The difference between the more and less hydrophobic compounds can be observed in the partitioning of the simulated PCDD/Fs at the Station 14 (Figure 8). The most hydrophobic compounds such as OCDD and HxCDF are mainly bound to particles while the less hydrophobic ones, for example TCDF, are distributed almost equally between the dissolved and particulate phase. However, phytoplankton blooms occurring mainly in spring produce a sudden increase of the DOC concentration in the system and the compounds’ distribution is affected by the high affinity to this fraction. As a consequence, peaks of DOC bound compounds are observed when blooms occur.

In general, the occurrence of peaks in the yearly simulation of the total water concentrations is consistent with rainfall events, suggesting the important role of wet deposition as an input of PCDD/Fs in the lagoon (Figure 8). Other studies have documented the importance of wet deposition for the input of PCDD/Fs, e.g., experimental measurements of dry and wet deposition fluxes in Houston [30] and the evaluation of global wet and dry deposition fluxes over the Atlantic Ocean reported in [31]. However, the role of wet deposition is likely to depend on the amount and frequency of rainfall that is recorded at a given site, and is therefore a regional attribute.

**Figure 9.**
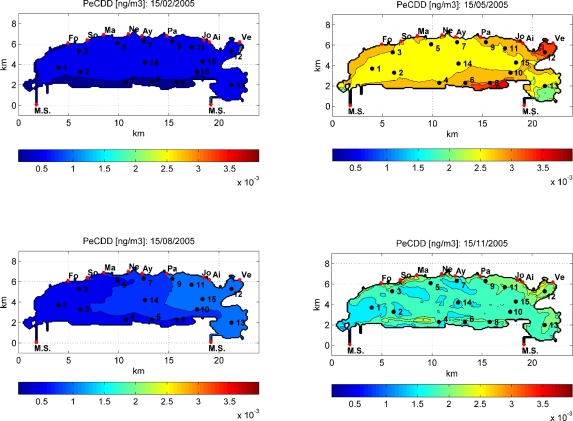
Spatial variability of the concentrations of PeCDD in Thau Lagoon during 2005.

Figure 9 illustrates, as an example, the model results about the seasonal spatial variability of PeCDD in Thau lagoon in February, May, August and November 2005. Higher concentrations are observed in May and November, corresponding to the peaks displayed in Figure 8, which basically could be attributed to rainfall input and/or ecosystem functioning, oppositely to the lower contamination and quite homogeneous spreading in February and August. The eastern part of the lagoon has lower water column contaminant concentrations, in proximity of Station 13, where it is connected to the Mediterranean Sea that brings less contaminated water. However, this part of the lagoon is the most urbanized and industrialized and historical contamination is also well reported for sediments. Nevertheless, since the model is forced with a homogeneous value for sediment concentrations, the simulations do not consider the effect of spatially heterogeneous sediment contamination.

The yearly average fluxes to and from the water column of Thau lagoon were calculated (Table 2). Positive fluxes represent inputs of contaminant from air or sediments to the water column, while negative fluxes stand for losses of contaminants, either to the airshed through volatilization or to the sediment through settling or within the water column by degradation. Results showed that the input of the PCDD/Fs considered in the simulations at the air-water interface is mainly driven by wet and dry deposition fluxes.

The sum of the total air-water fluxes of the simulated PCDD/Fs calculated from January 2005 to January 2006 is equal to 28 ng m^−2^ year^−1^ and it is in the same range as the measured average yearly fluxes measured in Venice lagoon from July 1998 to July 1999 [32], which are between 0.01 and 39.9 ng m^−2^ year^−1^ for the rural area, 5.22–49.1 ng m^−2^ year^−1^ for the urban area and 10.6–172 ng m^−2^ year^−1^ for the industrial area of Porto Marghera.

In general, the net input from atmosphere estimated by the model as a sum of volatilization, absorption, wet and dry deposition is positive except for TCDF, which compared to the other compounds is less chlorinated and shows higher volatilization potential. Therefore, volatilization is driving the air-water exchange for TCDF and on a yearly basis TCDF is released from the water to the atmosphere.

On the sediment-water interface the model estimates that the settling flux is a very important sink of PCDD/Fs and causes a loss of chemical that is higher than the input coming from the resuspension and diffusion fluxes. Similar results were found for the Venice lagoon [33] where a net deposition of PCDD/Fs from the air towards the sediments was estimated for most of the congeners, except TCDF, which was characterized by a net flux in the opposite direction.

**Figure 10.**
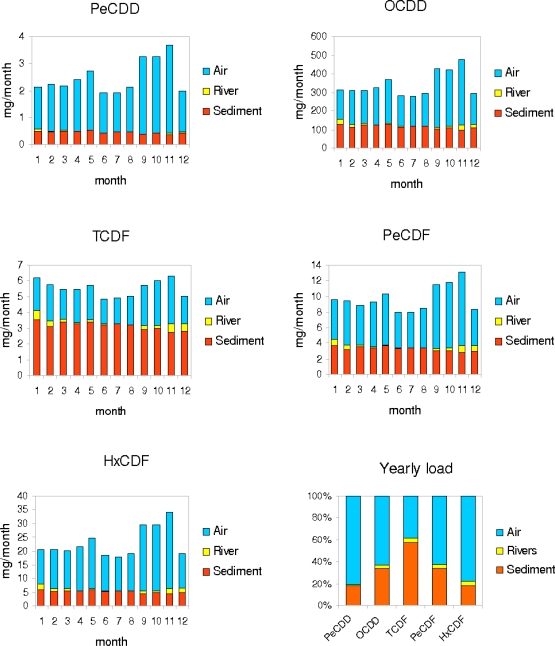
Simulated monthly (mg/month) and yearly (%) loads from atmosphere, sediments and riverine input for different PCDD/Fs congeners in the Thau lagoon.

The relative contribution of atmosphere, sediments and riverine input to the monthly and yearly balance of PCDD/Fs in the Thau lagoon is represented in Figure 10. For this purpose, only positive input fluxes were considered, thus for air it represents the sum of absorption, dry and wet deposition while for sediments it considers diffusion and resuspension.

The results emphasize the importance of atmospheric fluxes as a source of contamination for more chlorinated compounds as PeCDD, OCDD, PeCDF and HxCDF, while the loads of lighter ones - for example TCDF - are dominated by sediments. Oppositely, the input from rivers plays a minor role for all the considered contaminants.

The importance of atmospheric sources compared to sedimentary sources of PCDD/F has been documented for different marine environments, from open sea conditions, e.g., in the Baltic Sea [34], to coastal environments, e.g., along the Swedish coast of the Baltic Sea, where the source contribution of PCDD/F from the atmosphere varies between 60–80% [35] or in the Venice lagoon where about 60% of PCDD/Fs comes from the atmosphere [36]. The yearly loads predicted by our model are in agreement with these findings.

Finally, the model evaluation of monthly loads shows that atmospheric fluxes are generally more variable in time than sediment fluxes. This result is related to the variation of atmospheric inputs during wet deposition events, which seem to play a major role in the PCDD/Fs dynamics of the lagoon.

## 6. Conclusions

The present model results have highlighted the importance of the atmosphere as a relevant source of PCDD/Fs in the Thau lagoon water system.

Given the high affinity of the PCDD/Fs compounds to particles, the wet and dry deposition fluxes were found to be the major input fluxes for the system, and rainfall events caused an important increase of the contaminant concentration in the water column.

The results of the model emphasize the importance of monitoring not only water and sediments, but also the atmospheric compartment in order to assure good water quality. This should be considered in particular for ecosystems where the input of persistent organic pollutants, such as PCDD/Fs, may affect the biotic compartment, by bioaccumulation and biomagnification. For the Thau lagoon, reported concentrations of PCDD/Fs in mussels [27] are below the threshold set by the European Commission for fishery products for human consumption [37] and the trend from 1981 to 2005 shows a decrease of PCDD/Fs in marine mussels [38]. Nevertheless, the results pointed out the importance to implement policies for air quality at global scale that consider not only the direct effect on human health through inhalation, but also the indirect effect that may be caused by the atmospheric input of pollutants in an ecosystem such as a lagoon.

## Figures and Tables

**Table 1.** Concentration of PCDDs and PCDFs in different compartments used as forcing for the model, in ng m^−3^ [27].

	**Gas**	**Air Particulate**	**Rain**	**Sediment**	**River**	**Open Sea**
**PeCDD**	1.68·10^−6^	3.37·10^−6^	0.67	256	4 10^−3^	5 10^−4^
**OCDD**	3.00·10^−6^	3.80·10^−4^	75.99	54,784	1,842	5.7 10^−2^
**TCDF**	2.96·10^−6^	2.43·10^−6^	0.49	1,484	40 10^−3^	5 10^−4^
**PeCDF**	3.97·10^−6^	9.25·10^−6^	1.85	1,587	59 10^−3^	1 10^−3^
**HxCDF**	1.79·10^−6^	2.95·10^−5^	5.90	2,611	143 10^−3^	4 10^−3^

**Table 2.** Simulated fluxes of PCDDs and PCDFs to and out of the water column of Thau lagoon in ng m^−2^ year^−1^.

	**PeCDD**	**OCDD**	**TCDF**	**PeCDF**	**HxCDF**	**Σ PCDD/Fs**
Volatilization	−0.06	−0.06	−0.37	−0.31	−0.12	−0.92
Absorption	0.01	0.01	0.09	0.10	0.03	0.25
Wet deposition	0.07	7.73	0.05	0.19	0.59	8.63
Dry deposition	0.16	17.98	0.11	0.44	1.37	20.06
**Σ*Air–water Fluxes***	*0.19*	*25.65*	*−0.11*	*0.41*	*1.88*	*28.02*
Sedi/water diff.	0.01	1.75	0.04	0.05	0.07	1.91
Settling	−0.24	−50.31	−0.47	−0.88	−1.35	−53.25
Resuspension	0.05	12.05	0.33	0.35	0.39	13.18
**Σ*Sediment–water Fluxes***	*−0.19*	*−36.51*	*−0.09*	*−0.48*	*−0.89*	*−38.16*
